# The evolution of cancer therapies and their Implications for health technology assessment in Australia

**DOI:** 10.1186/s12962-026-00731-2

**Published:** 2026-03-12

**Authors:** Arun M. Jones, Stephen Goodall, Danny Liew, Hansoo Kim

**Affiliations:** 1 Health Economics Group, Griffith University, 1 Parklands Drive, Gold Coast, Queensland 4215 Australia; 2 Centre for Health Economics Research and Evaluation, University of Technology Sydney, Sydney, Australia; 3 Faculty of Health, Medicine and Behavioural Sciences, University of Queensland, Brisbane, Australia

## Abstract

Cutting edge cancer therapies are increasingly brought to market at high prices, creating a tension between getting new therapies to patients as quickly as possible, and doing so in a cost-effective manner. The earlier cancer therapies are granted market authorisation, the less mature data on efficacy, safety, and quality of life are. The resulting uncertainty can complicate health technology assessments (HTAs) and therefore delay reimbursement decisions, which the vast majority of patients rely on to gain access to expensive new medicines. This narrative review summarises recent innovations in cancer therapies from a health economic perspective in Australia. We describe how such therapies are financed on the Pharmaceutical Benefits Scheme and the challenges they bring to HTA, such as their increased reliance on surrogate endpoints, extrapolation of immature survival data, and high budget impact. We discuss current methods the field has developed to overcome these problems, including managed entry agreements, coverage with evidence development, early health economic modelling, and the use of real-world evidence. These strategies aim to balance innovation and affordability, ensuring that patients can access life-extending therapies while maintaining the sustainability of Australia’s healthcare system.

**Supplementary Information:**

The online version contains supplementary material available at 10.1186/s12962-026-00731-2.

## Background

The World Health Organization (WHO) has estimated that almost 10 million people died due to cancer in 2022 [1], and cancer is currently the leading cause of premature death in most developed countries in the world, including Australia [2]. The number of new cancer cases has grown steadily over the last four decades, with the Australian Institute for Health and Welfare (AIHW) reporting an 88% increase between 2000 and 2023. The age-standardised incidence was approximately 503 cases per 100,000 in 2022 [3]. Within the same period, the increase in age-standardised incidence for some cancers has been especially notable. The incidence of liver cancer increased by 114% from 4.2 to 9 per 100,000 and the incidence of small intestine cancer increased by 87% from 1.5 to 2.8 per 100,000. In 2023, it is estimated that 165,000 new cancer cases will be diagnosed and 51,000 people will die of cancer in Australia [4, 5].

Cancer is the leading cause of total disease and injury burden in Australia, as measured by years of healthy life lost from living with disease combined with years of life lost from premature death. According to AIHW estimates, cancer accounts for 16.6% of this burden, exceeding the burden due to mental and substance use disorders (14.5%), musculoskeletal disorders (12.8%), and cardiovascular diseases (11.8%) [6].

Cancer drugs are becoming more expensive in Australia [7], and the utilisation of cancer drugs is outpacing population growth [8]. Funding new cancer drugs therefore involves the careful balance between awarding innovation, providing timely access, and ensuring affordability for not only the public purse but also for patients and families. Unfortunately, there is now an increased reliance on earlier evidence to support funding decisions [9, 10], and it is therefore vital to establish new methodologies to cope with these issues.

## Evaluation of cancer therapies

The evaluation of cancer therapies focuses on four main groups of outcomes: clinical efficacy, safety, quality of life (QoL) and economic evaluation.

### Efficacy

Progression free survival (PFS) and overall survival (OS) are the most used efficacy endpoints in oncology clinical trials. OS is defined as the time from randomisation to the date of death from any cause. PFS is defined as the time between the date of randomisation and the first date of documented progression, based on radiological assessment of the cancer according to the response evaluation criteria in solid tumours (RECIST) [11], or death due to any cause.

### Safety

The safety of oncology therapies is reflected by adverse events (AEs), which are defined as any new untoward medical occurrence or worsening of a pre-existing medical condition in a patient receiving an investigational treatment. An AE can therefore be any unfavorable and unintended clinical or laboratory finding, or a disease temporally associated with the use of the investigational treatment, whether or not these are considered related to the investigational product. AEs are graded using the National Cancer Institute Common Terminology Criteria for Adverse Events [12]), starting from least severe with grade 1, to grade 5 which is one resulting in death.

### Quality of life

Quantity of life is naturally measured in life years, but quality of life (QoL) is a more difficult concept to measure in a useful way due to its subjective and multidimensional nature [13]. Questionnaires known as QoL instruments are often employed to measure the health of a patient. An example of such an instrument is the the European Organisation for Research and Treatment of Cancer (EORTC) Quality of Life Utility – Core 10 Dimensions (QLU-C10D) [14]. The QLU-C10D attempts to quantify health-related QoL by measuring different dimensions of health, such as physical functioning, role functioning, social functioning, emotional functioning, and areas more pertinent to cancer patients such as appetite loss, nausea, and bowel problems. The combination of responses given by a patient in each dimension of health included in the instrument makes up a health state.

Health related QoL instruments such as the QLU-C10D separates the human experience into discrete health states defined by the unique combination of responses possible within the instrument. We can then value each health state by assigning it a specific “utility” value on a continuous scale between 0 and 1, with a utility of 0 representing death and 1 representing perfect health.

These utility values are most often derived through the direct or indirect elicitation of preferences of the public and are hence often referred to as “preference-based measures”. In practice, this can mean describing health states as defined by an instrument, such as the QLU-C10D, to members of the public. Various techniques can then be used to derive a utility value for each described state.

When we multiply the time an individual spends in specified health states by each state’s associated utility weight, we gain a unit which values health outcomes, encompassing both the quantity and quality of life experienced by the individual. This is known as a quality-adjusted life year (QALY). For example, if we value a health state with a 0.7 utility weighting, then 10 years spent in that health state would be equivalent to 7 QALYs. QoL measures can therefore reflect the trade-offs between survival and several factors such as AEs of oncology treatments, treatment pathways, the cancer itself, and the stage of disease progression. As such, it is an important tool for the assessment of oncology therapies, as it gives us a common unit to measure the outcomes of any kind of medical intervention.

Many different QoL instruments exist, and some have been created specific to certain disease areas with the aim of capturing changes in QoL not normally reflected when more generic instruments are used. In the economic evaluation of cancer treatments, a common area of discussion is the use of condition-specific versus generic instruments [15–17], where a trade-off exists between the improved sensitivity of condition-specific instruments to certain aspects of QoL and their impaired comparability with generic instruments.

## Economic evaluation

The health sector, like any other, suffers from the fundamental economic problem of limited resources and unlimited wants. The funding of any health intervention comes at the cost of forgoing another. Economic evaluation is concerned with informing decision-makers as to the most efficient use of limited resources given the costs and consequences associated with specific strategies, and in the face of uncertainty surrounding the measurement of either of these.

Cost utility analysis is the most common form of economic evaluation used to inform decision makers. In cost-utility analysis, an economic model is developed to estimate the costs and outcomes associated with an intervention given the available evidence and assumptions about how the proposed intervention will work in practice versus a comparator (usually standard of care). The outcome of interest is the cost per additional QALY gained by a proposed intervention over the comparator. This ratio is known as the incremental cost-effectiveness ratio (ICER), which is compared against a pre-specified cost-effectiveness threshold (CET) value to determine whether a proposed intervention is worth the investment.

The cost-effectiveness threshold (CET) can be defined in two ways, the demand side definition operates under the assumption that a society has a certain amount it is willing to pay per extra unit of benefit (QALY) generated by a health intervention. If a proposed intervention is shown to produce additional QALYs at a cost less than our willingness to pay per QALY, then that intervention should then be funded on the grounds that it is cost-effective. The supply side CET is defined as the opportunity cost of health spending under the rationale that new interventions should only be funded if they generate QALYs at a lower cost per QALY than the marginal productivity of the health system overall [18].

Economic modelers can characterise the uncertainty associated with the model’s inputs and assess their impact on the ICER generated through sensitivity analysis, where model input variables are changed to see their impact on the resulting ICER. Generally, higher ICERs and greater uncertainty in the estimated ICER allows HTA agencies to negotiate lower prices. However, in areas with high unmet clinical need over standard of care, HTA agencies such as the PBAC are more likely to accept ICERs exceeding the CET [19].

## Overview of oncology therapies

Oncology therapies can be categorised as non-systemic or systemic. Non-systemic (localised) therapies mostly comprise radiotherapy and surgical interventions and are performed to gain local control of disease. Systemic treatment aims to limit distant spread of cancer or treat cancer that has already metastasized and will be the focus of this sub-section.

Systemic oncology therapy has evolved from antineoplastic chemotherapy to immunotherapies and targeted therapies over the past 20 years, with progressively improving efficacy. This is well-illustrated in systemic therapies for metastatic melanoma (Table 1), which initially involved chemotherapies such as hydroxyurea and dacarbazine, before targeted therapies and immune checkpoint inhibitors (ICIs) entered the scene in 2011 [20]. Newer therapies have been pivotal in increasing survival among patients with advanced melanoma. Balch et al. estimated the five-year survival for stage IV melanoma to be 10% in 2004 [22], while the AIHW estimated this figure to be 26% in 2019 [23].

**Table 1:** Chronology of the therapies approved to treat metastatic or unresectable stage III melanoma (1967–2022)

Treatment	FDA Approval Year	Type
Hydroxyurea	1967	Non-targeted
Dacarbazine	1975	Non-targeted
Interleukine-2	1998	Immunotherapy
Ipilimumab	2011	Immunotherapy
Vemurafenib	2011	Targeted
Dabrafenib	2013	Targeted
Trametinib	2013	Targeted
Pembrolizumab	2014	Immunotherapy
Nivolumab	2014	Immunotherapy
Nivolumab & ipilimumab	2015	Immunotherapy
Dabrafenib & trametinib	2015	Targeted
Vemurafenib & Cobimetinib	2015	Targeted
Talimogene laherparepvec	2015	Immunotherapy
Encorafenib & Binimetinib	2018	Targeted
Atezolizumab & Cobimetinib & Vemurafenib	2020	Immunotherapy and targeted
Relatlimab & Nivolumab	2022	Immunotherapy

Sources: Millet, A., et al.,*Metastatic Melanoma: Insights Into the Evolution of the Treatments and Future Challenges.*Medicinal Research Reviews, 2017.**37**(1): p. 98–148. [20, 21]. “Melanoma Treatments and Mortality Rate Trends in the US, 1975 to 2019.” JAMA Network Open 5 (12): e2245269.https://doi.org/10.1001/jamanetworkopen0.2022.45269[21]

### Chemotherapies

In general, chemotherapies inhibit cell proliferation and tumour multiplication by affecting either macromolecular synthesis or the function of neoplastic cells. This is achieved by interfering with DNA, RNA or proteins synthesis of the cancer, thereby preventing further invasion and metastases [24].

Chemotherapies are commonly associated with severe AEs as they also affect non-cancerous cells. Most chemotherapies target rapidly multiplying cells such as those in the gastrointestinal tract, skin, mucous membranes and bone marrow. Therefore, common AEs are constipation, nausea and vomiting, anorexia, mucositis and diarrhoea [25–27], as well as alopecia, drowsiness, fatigue and myelosuppression [28, 29]. AEs can be managed with other therapies, but these are often ineffective and do not address potential long-term sequelae [25]. Chemotherapy therefore poses a significant challenge as treatment decisions need to carefully balance benefits against significant AEs that may ultimately worsen the QoL of patients.

### Targeted therapies

Chemotherapies kill normal cells in addition to cancer cells and are therefore toxic to many organs, as discussed in the previous section. Therapies that can discriminate between normal and cancer cells, thus leading to less AEs, are desirable. This was the main motivation for the development of new targeted therapies that inhibit the growth of cancer cells. The idea of using cell growth inhibition in cancer treatments is far from new, with Ennis et al. publishing a review of the epidermal growth factor receptor (EGFR) system and tyrosine kinase inhibitors as antitumor therapy more than 30 years ago [30]. Targeted therapies inhibit kinases by stopping cell proliferation signaling [31]. There are many different kinase inhibitors affecting different kinases, for example, vascular endothelial growth factor receptor (VEGFR) is considered the master regulator of angiogenesis during growth and development, as well as in disease states such as cancer, diabetes and macular degeneration [32]. Sorafenib and sunitinib are examples of drugs that inhibit VEGFR. Another tyrosine kinase that has been well studied is the EGFR, which is an enzyme that spans the cell membrane, with one end of the protein projecting from the cell surface. Gefitinib and lapatinib are examples of compounds that inhibit EGFR [33].

Targeted therapies have revolutionised cancer treatment in several ways. First, many of these drugs are small molecules that are administered orally, thereby saving patients time and costs of attending an infusion at a hospital or outpatient clinic. Secondly, these drugs may slow progression of disease more effectively than chemotherapies. Erlotinib, one of the first targeted therapies developed to treat non-small cell lung cancer (NSCLC), was shown to significantly delay disease progression in patients with mutation of EGFR signaling pathway in clinical trials. A meta-analysis by Lee et al. [34] estimated the PFS hazard ratio for erlotinib versus chemotherapy to be 0.43 (95% CI: 0.38 to 0.49, *p*-value 0.001). Significant improvement in PFS is a common observation for targeted therapies. The observed hazard ratios for PFS of eight different targeted therapies for the treatment of advanced melanoma, gastrointestinal stromal tumours, hepatocellular carcinoma, NSCLC, RCC and soft tissue sarcoma are listed in Table 2. They vary from 0.30 (95% CI: 0.18 to 0.51) for dabrafenib versus dacarbazine for the treatment of advanced melanoma [35] to 0.58 (95% CI: 0.42 to 0.80) for nilotinib vs best supportive care for the treatment of Gastrointestinal stromal tumours [35, 36]. Common to all these examples is that the hazard ratio is statistically significantly lower than 1.0 for targeted therapies compared to the standard of care of the time.

**Table 2:** Hazard ratios in progression free survival (PFS) for targeted vs non-targeted therapies

Comparison	Hazard Ratio (PFS)	Indication
dabrafenib vs dacarbazine	0.30 (95% CI 0.18 to 0.51) [35]	Advanced melanoma
nilotinib vs best supportive care	0.58 (95% CI: 0.42 to 0.80) [36]	Gastrointestinal stromal tumours
sunitinib vs interferon-alpha	0.42 (95% CI: 0.32 to 0.54) [37]	RCC
cabozantanib vs placebo	0.44 (95% CI: 0.36 to 0.52) [38]	HCC
crizotinib vs chemotherapy	0.49 (95% CI: 0.37 to 0.64) [39]	NSCLC (ALK+)
erlotinib vs chemotherapy	0.37 (95% CI: 0.25 to 0.54) [40]	NSCLC (EGFR+)
everolimus vs placebo	0.30 (95% CI: 0.22 to 0.40) [41]	RCC
pazopanib vs placebo	0.31 (95% CI: 0.24 to 0.40) [42]	Soft tissue sarcoma

ALK+: anaplastic lymphoma kinase positive, AM: advanced melanoma, BRAF+: B-Raf serine-threonine kinase, EGFR+: epidermal growth factor receptor positive, GIST: gastrointestinal stromal tumours, HCC: hepatocellular carcinoma, NSCLC: non-small cell carcinoma, RCC: renal cell carcinoma.

The third advantage of some targeted therapies over chemotherapy is a better safety profile. Common AEs are gastrointestinal and dermatological in nature, with cutaneous adverse reactions occurring especially with TKIs [43–46]. For EGFR inhibitors such as erlotinib and gefitinib, this is due to inhibition of EGFR activity, leading to a cascade of cellular events resulting in multiple cutaneous AEs, including rash, dry skin, pruritus and inflammation of the nail or periungual tissues [47]. A review by Livingstone et al. [46] found that AEs for BRAF and MEK inhibitors primarily affected the skin and the severity of the AEs were generally mild to moderate. Another review [44] found that VEGFR inhibitors such as pazopanib and sorafenib were associated with similar AEs. The most common (reported by more than 25% of patients) AEs (any grade) for EGFR inhibitors, BRAF/MEK inhibitors and VEGFR inhibitors are displayed in Table 3.

**Table 3:** ICIs TGA approvals, PBS listing dates and prices

Drug	#TGA approval **(1**^**st**^**indication)**	*Listed on the PBS **(1**^**st**^**submitted)**	*Dispensed price maximum amount in public hospitals	Indications
Atezolizumab	July 2017 (Non-small cell lung cancer)	April 2018 (July 2017)	$6,838.60	Urothelial cancer Small cell lung cancer Liver cancer
Avelumab	January 2018 (Merkel cell carcinoma)	May 2019 (March 2018)	$7,828.17	Merkel Cell Carcinoma Urothelial cancer
Durvalumab	October 2018 (Urothelial carcinoma)	March 2020 (July 2018)	$10,853.55	Non-small cell lung cancer
Ipilimumab	June 2011 (Melanoma)	August 2013 (March 2011)	$16,125.09	Non-small cell lung cancer Renal cell carcinoma
Nivolumab	January 2016 (Melanoma)	May 2016 (March 2015)	$9,560.79	Adjuvant melanoma Non-small cell lung cancer Renal cell carcinoma Head and neck cancer Relapsed/refractory Hodgkin’s’ lymphoma Urothelial cancer
Pembrolizumab	April 2015 (Melanoma)	September 2015 (November 2014)	$7,891.46	Adjuvant melanoma Colorectal cancer refractory primary mediastinal large B-cell lymphoma Relapsed/refractory Hodgkin’s’ lymphoma Head and neck cancer Non-small cell lung cancer
Cemiplimab	July 2020	November 2021 (November 2020)	$7,381.23	Cutaneous squamous cell carcinoma Non-small cell lung cancer
Dostarlimab	February 2022	November 2023 (March 2022)	$7,381.23	Endometrial carcinoma

Sources:http://www.tga.gov.au, *http://www.pbs.gov.au

Although having a better safety profile than chemotherapy, QoL is still often impacted by targeted therapies [48]. Indeed, the number of QoL questionnaires developed has grown in line with the emergence of targeted therapies for specific cancers. For example, the functional assessment of cancer therapy (FACT) published a kidney version in 2007 [49] in line with sunitinib being developed. This was followed by a FACT instrument for melanoma in 2009 [50] as vemurafenib and dabrafenib were developed. Moreover, mapping of existing general cancer questionnaires such as QLQ-C30 are common [51–55].

Unfortunately, targeted therapies have not proven to be a long-term solution for cancer patients. While disease progression is delayed, very few patients experience complete response as per the RECIST criteria. For example, in the RCC trial presented above [37], no patients experienced a complete response (both sunitinib and interferon-alpha treatment arms) and in the advanced melanoma trial [35], 3% of dabrafenib-treated patients experienced a complete response versus 2% for dacarbazine-treated patients. In some instances, this has also translated into a lack of OS benefit.

At present, there are 46 targeted therapies available on the PBS, divided into 13 different classes [56] (Supplement 2). The PBS lists reimbursement prices in the form of Dispensed Price for Maximum Quantity (DPMQ) for each drug. DPMQs are generally representative of government outlays for older, off-patent drugs (Formulary 2 drugs), but for newer drugs who are still under patent (Formulary 1), DPMQ is a poor indicator of net government cost since generally these are subject to commercial-in-confidence rebates or risk-sharing agreements which reduce the true cost to the government from this published price.

The DPMQ of targeted therapies range from $30,225.12 for asciminib to $149.09 for imatinib, with only six of these 45 drugs having a DPMQ of less than $1000 (Supplement 3). Treatment costs are therefore often considerably higher than chemotherapy, with monthly drug costs of between $5,000 to $10,000 [57]. It has been estimated that cancer treatment costs grew more than 40% from 2009 to 2012 for the government due to the introduction of targeted therapies [58], which highlights the issue with funding and value for money as outlined previously.

### Immune checkpoint inhibitors

The introduction of ICIs for the treatment of cancer marked a shift away from targeting cells to utilising the immune system to fight cancer. The concept is to disrupt the signaling pathways that allow cancer cells to evade T-cell-mediated death. This is achieved by preventing cytotoxic T lymphocyte-associated protein 4 (CTLA-4) and programmed cell death protein 1 (PD1) from binding with specific ligands, thereby enhancing the immune system’s ability to attack malignant cells [59]. There are currently three different types of ICIs used to treat cancer in Australia; CTLA-4, PD-1 and programmed cell death protein ligand 1 (PD-L1) inhibitors. CTLA-4 is normally expressed on the surface of naïve effector T-cells, which inhibit autoimmunity, and promotes tolerance to selfantigens. Similarly, PD1 is an immune inhibitory receptor which negatively regulates T-cell functions through the engagement of programmed cell death protein ligand 1 PDL1, which is found on varies malignant cells [60].

The first ICI approved by the Australian Therapeutics and Goods Administration (TGA) was ipilimumab for treatment of melanoma in 2012 (Table 3). This was followed by pembrolizumab and nivolumab in 2015, also for melanoma. Subsequently, atezolizumab (NSCLC), avelumab (Merkel cell carcinoma), durvalumab (urothelial carcinoma), cemiplimab (cutaneous squamous cell carcinoma), and dostarlimab (endometrial carcinoma) were approved in 2017, 2018 and 2019, respectively. Government subsidisation of each ICI via the PBS followed between nine and 38 months after registration. Currently, eight ICIs are indicated for many different tumour types, including metastatic melanoma, colorectal cancer, NSCLC, metastatic urothelial cancer and RCC. Their DPMAs range from $6,838 to $16,125.

The efficacy and safety profiles of the ICIs differ substantially from those of chemotherapies and targeted therapies. In general, they demonstrate superior PFS and OS over standard of care (SoC), whether that compromises chemotherapies, placebo, targeted therapies or any combination of these.

ICIs often need time to activate the immune system before they have an impact on the cancer [61]. This is evident in various clinical trials as shown by the delay in separation of the PFS curves for ICIs compared to comparators [62–65]. As a result, no difference is occasionally reported for PFS despite a significant OS gain being observed which is especially the case when a targeted therapy is the comparator.

An example of this can be found in a randomised controlled trial of RCC patients treated with the PD-1 inhibitor nivolumab versus the targeted therapy everolimus [66]. The HR for PFS was observed to be not statistically significant, at 0.88 (95% CI: 0.75; 1.03), in contrast to the HR for OS, at 0.73 (98.5% CI: 0.57; 0.93). However, updated five-year data from the same study found PFS benefits to be statistically significant, with a HR of 0.84 (95% CI: 0.72; 0.99) [67].

Another example is nivolumab for the treatment of squamous cell carcinoma of the head and neck, the trial for which showed that PFS was not significantly better than a collection of comparators, with a HR of 0.89 (95% CI: 0.70 − 1.13), even though OS was superior (HR = 0.70, 97.73% CI: 0.51 to 0.96). The collection of comparators included cetuximab, a targeted therapy.

An interesting characteristic of OS curves in ICI trials is that they often plateau, implying the potential for long term survival. For example, in the CheckMate066 trial of nivolumab versus dacarbazine in patients with advanced melanoma [62], the OS curve for nivolumab only dropped 10% (from 80% to 70%) between seven and 17 months. In the same period, the OS for dacarbazine dropped 45% (from 65% to 20%). Only 7.6% of the nivolumab-treated patients had a complete response.

Safety profile also differs between ICIs and other systemic therapies. ICIs are associated with immune-related adverse events (irAE), which occur due to non-specific activation of the immune system [68].

Trial data for ipilimumab in advanced melanoma showed that almost all patients (98.4%) reported an AE (any grade) and 45% reported grade 3 or 4 AEs [69]. AEs specific to ipilimumab in the trial were life-threatening toxic epidermal necrolysis, colitis, hypophysitis, hepatitis, pancreatitis, iridocyclitis, lymphadenopathy, neuropathies, and nephritis. While the rate for AEs of any grade for other ICI treatments in advanced melanoma is also high (nivolumab =93.2% [62], pembrolizumab = 79.5% [70]), the rate of grade 3 or 4 AEs is much lower (nivolumab = 4.0% [62], pembrolizumab = 13.3% [70]).

There is evidence that these rates are higher in a real-world setting. Indeed this was the concern of the South Australian Medicines Evaluation Panel (SAMEP), which submitted a letter to the PBAC [71]. The letter contained data on the incidence and the costs of treating AEs associated with ipilimumab. Specifically, eight ipilimumab-treated patients were admitted to hospital and received infliximab for severe, steroid-refractory colitis secondary to ipilimumab. Treatment of this particular irAE for these patients was estimated to cost between $395,976 and $483,840, thereby adding $50,000–$60,000 to the total cost of ipilimumab per patient.

Treatment of irAEs has become increasingly important as ICI-treated patients are surviving longer with cancer. Treatment guidance and experience with irAEs is a major focus of ICI treatments [72–75]. New South Wales guidelines outline eleven categories of irAEs and how to manage them [76]. These are graded in the same way as standard AEs, on a scale of one to four. Various toxicities were identified in connection with ipilimumab treatment for melanoma [77], including cardiotoxicities, haematological toxicities, ocular toxicities and thyroid toxicities which were not widely reported for ipilimumab in clinical studies.

A systematic review by Abdel-Rahman et al. showed that the use of PD-L1 inhibitors was associated with an improvement in QoL across a variety of solid tumours using standard tools such as QLQ-C30 and FACT [78]. A systematic review by Hall et al. examined nivolumab, pembrolizumab, atezolizumab and ipilimumab across several cancer types and found that ICIs are well-tolerated with respect to QoL [79]. Similar to Abdel-Rahman et al., the instruments were QLQ-C30 and FACT, but this review also included evaluation of EQ-5D. A recent review by Faury et al. is highly critical of the review reported by Abdel-Rahman et al. and Hall et al., arguing that the QoL tools currently available have not been validated in cancer patients treated with ICIs [80]. This is especially important when assessing the impact of irAEs. For example, in the clinical trial investigating nivolumab for squamous cell carcinoma of the head and neck, the Quality of Life Questionnaire for Head and Neck cancer 35-items (QLQL-H&N35) was used [81]. In the trial, 7.6% of nivolumab-treated patients reported rash and 7.2% reported pruritus, but the QLQ-H&N35 does not assess skin problems.

### Chimeric antigen receptor cell therapies

Since immunotherapies, the latest approved class of drugs showing great promise in the treatment of cancer are chimeric antigen receptor (CAR) T-cell therapies. These therapies involve the collection of patients’ own T-cells, which are modified and then reinserted into the patient with an enhanced ability to detect and kill cancer cells.

The first CAR T cell therapy approved by the FDA was tisagenlecleucel in 2017 [82], whose application was granted Priority Review, Breakthrough Therapy Designation, and Orphan Product Designation [83]. Priority Review and Breakthrough Therapy Designation both act to speed up the FDA review process due to unmet clinical need, whilst Orphan Product Designation provides extra incentives to drug-makers to create drugs in rare disease areas which would otherwise not be commercially attractive.

Whilst CAR-T cell therapies have resulted in long-term clearance in otherwise refractory haematological malignancies. They are associated with severe adverse events, require highly personalised manufacturing, and have found little success in treating solid tumours [82, 84, 85]. The ‘next generation’ of CAR therapies aim to overcome these problems, with CAR natural killer therapies in early-stage clinical trials. So far, none have gained market authorisation.

### Shifting evidence requirements for market authorisation

The introduction of TKIs, ICIs and CAR-T cell therapies brought with it a new paradigm of generating clinical evidence. Where previously randomised clinical trials were powered to estimate relative treatment effects in populations based on their tumour site of origin, the advent of precision cancer medicine increased the prevalence of treatments where the treatment population was instead selected based on genomic, biologic and/or immune markers [86]. In medicines with rarely expressed such targets, it may be too difficult or infeasible to find enough patients to power a typical phase III clinical trial. In place of this, regulators may instead grant approval on the basis of trials with very small sample sizes or even single-arm trials [87]. There has also been a significant growth in the prevalence of immature OS data in pivotal trials used to gain regulatory approval. Clinical trials in melanoma are highly illustrative of this issue, earlier trials in metastatic melanoma such as ipilimumab’s [69] had overall survival as the primary endpoint, whereas later trials had PFS as a primary endpoint, with OS as a secondary endpoint [88]

In addition to the challenges associated with small sample sizes and immature data, advanced cancer treatments have in some cases been granted market authorisation at a lower standard of evidence as a result of their strong early promise over standard of care precluding randomised clinical trials from taking place on ethical grounds. In cases where a new therapy is so clearly an improvement over standard of care, it becomes unethical to randomise patients between treatment and control arms. One example of this is the case of imatinib for the treatment of chronic myeloid leukemia [89], where early evidence made it clear that treatment with imatinib was superior to natural history. The natural history of the disease was well known, and thus the FDA granted market authorisation based on a single-arm trial. Similarly, a later RCT of imatinib [90] then allowed patients in the standard of care arm to crossover into the treatment arm once intolerance to initial treatment or lack of efficacy could be demonstrated. The usage of non-randomised [91], single arm trials [92], which allow treatment switching [93] are all becoming increasingly common in new cancer drugs granted market authorisation today, at the cost of adding difficulty and uncertainty to the estimation of their relative treatment effects.

## Financing cancer treatments in Australia

Funding cancer medications is a challenge as mentioned in previous sections. The price of oncology drugs makes it impossible for most patients to pay out of their own pockets. Instead, patients rely on the PBS for access to these medicines. However, public funding of medications for cancer is a challenge when compared to other conditions. This is due to a combination of limited evidence available (e.g. immature survival, surrogate endpoints etc.) and the high price of the medications.

The PBS is a government-subsidised scheme that aims to provide affordable and equitable access to necessary medicines for Australians [94]. Medicines dispensed via the PBS come with a standard out-of-pocket co-payment of $31.60 [56]. From July 2023 to June 2024, 223 million prescriptions were dispensed through the PBS, from a list of more than 900 different medicines [95]. This accounted for $17.4 billion in government expenditure, which equated to 16.6% of the direct healthcare budget of $76.15 billion in that year [96]. Of the top three most costly drugs on the PBS, two were ICIs: nivolumab ($333,970,002) and pembrolizumab ($331,481,924).

The Australian health care budget is set to rise from $112.7 billion in 2024/25 to 122.7 billion in 2027/28. Government projections estimate that pharmaceutical benefits spending is expected to fall in real terms over the same time period as a result of “existing pricing policies” [97], likely in reference to price disclosure and the various statutory price reduction mechanisms in place for medicines whose patent has expired [98]. However, due to their novelty, cutting-edge cancer treatments such as those discussed in this paper do not fall under the scope of such policies. For example, all eight ICIs currently reimbursed on the PBS are still patented, and given that usage of ICIs is likely to increase over time [99, 100], the proportion of PBS expenditure they consume will only rise with it.

All medicines, including cancer drugs, undergo a comprehensive HTA process before being listed on the PBS. The process starts with a sponsor (usually a pharmaceutical company) submitting an application for the listing of the drug in question. The submission is assessed by the PBAC, which makes recommendations to the Commonwealth Department of Health and the Federal Minister of Health [101]. The PBAC is comprised of independent clinicians, other healthcare professionals, health economists, consumer representatives and an industry-nominated member. One-third of the members are nominated by the Health Minister and the others by professional organisations. Ultimately, the Health Minister determines which drugs are listed on the PBS, based on the advice of the PBAC.

### Challenges for health technology assessment in oncology

Critical to the estimation of the benefits and costs associated with different interventions is the availability of good quality evidence on which to base these estimates. Cancer drugs are often placed on accelerated market approval pathways with the intention of getting potentially lifesaving care to patients as soon as possible [102]. The TGA offers accelerated approval pathways, most notably the provisional approval pathway, akin to the FDA’s accelerated approval pathway. These pathways are for new medicines which meet unmet needs and speed up approvals by accepting surrogate outcomes from preliminary clinical data, usually phase II clinical trials or interim phase III clinical trial results. In these pathways, regulators accept surrogate outcomes, which are outcomes that are “reasonably likely” to predict traditional clinical outcomes (e.g. overall survival). This temporarily circumvents the need to wait for primary endpoints in longer phase III clinical trials to mature in favor of these surrogates, which mature much faster. Typical surrogate outcomes include PFS, objective response rate (tumor shrinkage), or even changes in biomarkers amongst patients. These accelerated pathways are generally conditional, granting only a time-limited marketing authorisation, which is subject to later confirmatory trials to verify this benefit after which the conditional marketing authorisation may be converted to a standard authorisation.

Since accelerated approval pathways do not require surrogates to be validated (proven to reliably predict OS), the evidence left to guide reimbursement decisions is often limited and uncertain. Indeed, accelerated approval pathways have been shown to have a lower standard of evidence for treatment effectiveness [103, 104]. A retrospective analysis of FDA cancer drugs approved based on surrogate endpoints found that only 16% made a level 1 (most robust) validation of the surrogate endpoint used, whilst in traditional approvals, 50% conducted a level 1 analysis [105]. Based on drugs with at least 5-years of follow Between 2013 and 2017, only 43% of those granted accelerated approval went on to demonstrate clinical benefit in confirmatory trials [106].

In recognition of the increasing use of surrogate outcomes to inform reimbursement decisions, a working group comprised of international HTA agencies published a whitepaper issuing guidance on the validation of surrogate endpoints specifically for use in reimbursement decisions [107]. Whilst this guidance has been successful in addressing the previously fragmented advice on how surrogate outcomes should be considered for decision making, the report does not address the issue of medicines who are granted accelerated approval often being in a situation where validation of the endpoint is not even possible due to its novelty. Unfortunately, in many cases, no data exists for the surrogate to be validated against.

Reimbursement assessments rely on evidence that often require translation and extrapolation. One important reason for this is that clinical trials typically do not provide data required for HTAs, since they usually aim only to demonstrate a favourable risk/benefit profile, for which short-term outcomes are often adequate. This leads to uncertainty with respect to cost-effectiveness and value for money as outcomes in many instances need to be transformed or translated to suit a local patient population with different profile than the one examined in the clinical trial. This is usually the case with respect to QoL, as most clinical trials are reported using non-Australian weights.

Another major problem is the extrapolation of premature survival data well beyond the trial duration. In some instances, cost-effectiveness is dependent on patients surviving more than 10–20 years into the future in order for the medications to be deemed cost-effective. Individual patient data may be parametrically extrapolated to estimate survival data over this time period, although the choice of parametric model to fit the data can result in widely varying results [108]. The choice of method for extrapolating OS has been shown to significantly affect predicted outcomes specifically in immuno-oncology [109].

In addition to the increased use of surrogate outcomes and extrapolation of immature survival data, the increased prevalence of single arm trials and treatment crossovers in the pivotal trials for new cancer medicines, combined with their high prices, all act to increase uncertainty surrounding the ICER generated in their economic evaluations. Whilst a new medicine might be granted market authorisation, a high degree of uncertainty surrounding the ICER makes the PBAC less likely to recommend it for reimbursement [110]. HTA agencies are put under significant political and social pressure when cutting-edge cancer medicines with regulatory approval fail to receive positive reimbursement recommendations [111, 112].

The lack of robust evidence resulting from issues such as these has been identified as a challenge specifically in the economic evaluation of cancer therapies [113, 114]. Counterproductively, accelerated market approval pathways can therefore slow down reimbursement decisions which rely on economic evaluations [115, 116]. This slows patient access to new drugs since, given the high price of cancer therapies, reimbursement is usually a prerequisite for access to these new treatments for the average patient. This lack of mature data is one of the biggest challenges in the HTA of cancer therapies currently. The discipline is actively developing various methodologies to supplement immature trial data, such as the use of managed entry agreements, real-world evidence, early economic modelling, and value of information analysis [117, 118].

Managed entry agreements (MEAs) are formal arrangements between payers and manufactures with the aim of sharing the financial risk due to uncertainty surrounding the introduction of new technologies [119, 120]. A high proportion of MEAs are used for new cancer drugs [121], and can generally be categorised as either finance-based agreements (FBAs) or performance-based agreements (PBAs) [122]. FBAs aim to share risk between payers and sponsors by implementing budget/volume caps, price-volume agreements, rebates, patient-level cost caps, whilst PBAs either make future reimbursement conditional on the generation of further evidence (coverage with evidence development), or link outcomes directly to payment through post-marketing clinical registries.

PBAs are generally seen as being associated with more barriers to use than FBAs [123]. In most health systems, including Australia’s, FBAs are more common due to their perceived simplicity [124–126]. Whilst managed entry with evidence development has been shown to successfully reduce uncertainty surrounding outcomes and result in positive PBAC recommendations [120], real world evidence generated by clinical registries such as those created for performance based MEAs is still limited in its ability to effectively inform reimbursement decisions due to the absence of control groups, non-standardised data collection and analysis, and high risk of bias and confounding when compared to RCTs [127].

Early HTA is increasingly being used as a method to help mitigate the impact of ICER uncertainty on reimbursement decisions [128]. Early HTA may be defined as “all methods used to inform industry and other stakeholders about the potential value of new medical products in development, including methods to quantify and manage uncertainty” [129]. Early economic modelling allows sponsors to characterise uncertainty surrounding the cost effectiveness of new treatments much earlier than what would be typical. This means sponsors and stakeholders can identify areas where evidence is lacking, informing future trial design and potentially generate evidence which reduces the uncertainty associated with HTA before it becomes time to apply for reimbursement. Value of information (VOI) analysis can help sponsors identify whether such research is worth conducting and help to design MESs. Although VOI analysis has been observed to inform reimbursement decisions [130], little empirical evidence exists on the effectiveness of early HTA and VOI in managing uncertainty created by accelerated approvals and improving the chance of first-time reimbursement recommendations.

These topics are now at the forefront of health economic and outcomes research. In its Top 10 health economics and outcomes trends 2024–2025 report, The Professional Society for Health Economics and Outcomes Research (ISPOR) listed real-world evidence at number one, drug pricing at two, and accelerated approvals at number six, underscoring the importance of generating methodologies that can support reimbursement decisions in the face of shrinking evidence bases. We can expect these areas to play a central role in health economic research to come.

## Electronic supplementary material

Below is the link to the electronic supplementary material.

Supplementary material 1

Supplementary material 2

Supplementary material 3

## Data Availability

No datasets were generated or analysed during the current study.
