# Supplementary material for: The evolution of cancer therapies and their Implications for health technology assessment in Australia
Source: Cost Eff Resour Alloc. 2026 Mar 12;24:52. doi: 10.1186/s12962-026-00731-2 (PMC13097878; doi:10.1186/s12962-026-00731-2)
Supplement: Supplementary file 1 — Supplementary material 1 [file 12962_2026_731_MOESM1_ESM.docx]

**Supplement 1: Antineoplastic agents dispensed on the PBS in 2022**

abemaciclib

acalabrutinib

afatinib

alectinib

arsenic

atezolizumab

avelumab

axitinib

azacitidine

bendamustine

bevacizumab

binimetinib

bleomycin

blinatumomab

bortezomib

brentuximab vedotin

brigatinib

busulfan

cabazitaxel

cabozantinib

capecitabine

carboplatin

carfilzomib

carmustine

celecoxib

cemiplimab

ceritinib

cetuximab

chlorambucil

cisplatin

cobimetinib

crizotinib

cyclophosphamide

cytarabine

dabrafenib

daratumumab

dasatinib

docetaxel

doxorubicin

doxorubicin - pegylated liposomal

durvalumab

elotuzumab

encorafenib

entrectinib

epirubicin

eribulin

erlotinib

etoposide

everolimus

fludarabine

fluorouracil

fotemustine

gefitinib

gemcitabine

gemtuzumab ozogamicin

gilteritinib

hydroxycarbamide

ibrutinib

idarubicin

idelalisib

ifosfamide

imatinib

inotuzumab ozogamicin

ipilimumab

irinotecan

lapatinib

larotrectinib

lenvatinib

lorlatinib

melphalan

mercaptopurine

midostaurin

mitozantrone

nilotinib

nintedanib

niraparib

nivolumab

obinutuzumab

ofatumumab

olaparib

osimertinib

oxaliplatin

paclitaxel

palbociclib

panitumumab

pazopanib

pembrolizumab

pemetrexed

pertuzumab

ponatinib

pralatrexate

raltitrexed

ribociclib

ripretinib

rituximab

ruxolitinib

sacituzumab govitecan

selinexor

sonidegib

sorafenib

sunitinib

temozolomide

tepotinib

tioguanine

topotecan

trametinib

trastuzumab

trastuzumab emtansine

vemurafenib

venetoclax

vinblastine

vincristine

vinorelbine

vismodegib

vorinostat

zanubrutinib
