# Supplementary material for: The evolution of cancer therapies and their Implications for health technology assessment in Australia
Source: Cost Eff Resour Alloc. 2026 Mar 12;24:52. doi: 10.1186/s12962-026-00731-2 (PMC13097878; doi:10.1186/s12962-026-00731-2)
Supplement: Supplementary file 2 — Supplementary material 2 [file 12962_2026_731_MOESM2_ESM.docx]

**Supplement 2: Targeted therapies on the PBS as of 07 March 2025**

| L01EA - BCR-ABL tyrosine kinase inhibitors |
| --- |
| ASCIMINIB |
| DASATINIB |
| IMATINIB |
| NILOTINIB |
| PONATINIB |
| L01EB - EGFR tyrosine kinase inhibitors |
| AFATINIB |
| ERLOTINIB |
| GEFITINIB |
| OSIMERTINIB |
| L01EC - BRAF (a gene that encodes the protein B-Raf) inhibitors |
| DABRAFENIB |
| ENCORAFENIB |
| VEMURAFENIB |
| L01ED - Anaplastic lymphoma kinase (ALK) inhibitors |
| ALECTINIB |
| BRIGATINIB |
| CERITINIB |
| CRIZOTINIB |
| LORLATINIB |
| L01EE - Mitogen-activated protein kinase (MEK) inhibitors |
| BINIMETINIB |
| COBIMETINIB |
| SELUMETINIB |
| TRAMETINIB |
| L01EF - Cyclin-dependent kinase (CDK) inhibitors |
| ABEMACICLIB |
| PALBOCICLIB |
| RIBOCICLIB |
| L01EG - Mammalian target of rapamycin (mTOR) kinase inhibitors |
| EVEROLIMUS |
| L01EH - Human epidermal growth factor receptor 2 (HER2) tyrosine kinase inhibitors |
| LAPATINIB |
| L01EJ - Janus-associated kinase (JAK) inhibitors |
| RUXOLITINIB |
| L01EK - VEGFR tyrosine kinase inhibitors |
| AXITINIB |
| L01EL - Bruton's tyrosine kinase (BTK) inhibitors |
| ACALABRUTINIB |
| IBRUTINIB |
| ZANUBRUTINIB |
| L01EM - Phosphatidylinositol-3-kinase (Pi3K) inhibitors |
| IDELALISIB |
| L01EX - Other protein kinase inhibitors |
| CABOZANTINIB |
| ENTRECTINIB |
| GILTERITINIB |
| LAROTRECTINIB |
| LENVATINIB |
| MIDOSTAURIN |
| NINTEDANIB |
| PAZOPANIB |
| RIPRETINIB |
| SELPERCATINIB |
| SORAFENIB |
| SUNITINIB |
| TEPOTINIB |
