# Supplementary material for: The evolution of cancer therapies and their Implications for health technology assessment in Australia
Source: Cost Eff Resour Alloc. 2026 Mar 12;24:52. doi: 10.1186/s12962-026-00731-2 (PMC13097878; doi:10.1186/s12962-026-00731-2)
Supplement: Supplementary file 3 — Supplementary material 3 [file 12962_2026_731_MOESM3_ESM.docx]

**Supplement 3: Targeted therapies and their prices on the PBS (March 2025)**

| Legal Instrument Drug | DPMQ (AUD) |
| --- | --- |
| abemaciclib | 4250.45 |
| acalabrutinib | 7414.57 |
| afatinib | 2750.40 |
| alectinib | 6483.03 |
| asciminib | 6175.10 |
| asciminib | 6131.79 |
| asciminib | 30225.12 |
| axitinib | 1070.79 |
| axitinib | 4950.61 |
| binimetinib | 7396.83 |
| brigatinib | 6815.68 |
| brigatinib | 6815.710 |
| cabozantinib | 9472.60 |
| ceritinib | 6934.49 |
| cobimetinib | 7035.11 |
| crizotinib | 6934.49 |
| dabrafenib | 4826.09 |
| dabrafenib | 7157.83 |
| dasatinib | 1322.75 |
| dasatinib | 828.74 |
| dasatinib | 1622.10 |
| encorafenib | 7035.15 |
| encorafenib | 5303.46 |
| entrectinib | 7290.90 |
| erlotinib | 528.55 |
| erlotinib | 651.87 |
| erlotinib | 151.76 |
| everolimus | 179.99 |
| everolimus | 303.86 |
| everolimus | 324.69 |
| everolimus | 307.87 |
| everolimus | 530.42 |
| everolimus | 560.31 |
| everolimus | 872.29 |
| everolimus | 1736.15 |
| everolimus | 1591.24 |
| everolimus | 1639.91 |
| everolimus | 1150.77 |
| everolimus | 2284.31 |
| everolimus | 2121.68 |
| everolimus | 2170.35 |
| everolimus | 1738.38 |
| everolimus | 458.19 |
| everolimus | 901.84 |
| everolimus | 368.15 |
| everolimus | 547.99 |
| everolimus | 901.63 |
| gefitinib | 536.84 |
| gilteritinib | 25145.6 |
| ibrutinib | 7954.15 |
| ibrutinib | 10551.33 |
| ibrutinib | 5356.97 |
| idelalisib | 5119.46 |
| imatinib | 149.09 |
| imatinib | 289.69 |
| imatinib | 560.09 |
| lapatinib | 2210.49 |
| larotrectinib | 10062.60 |
| larotrectinib | 2637.60 |
| larotrectinib | 3462.60 |
| lenvatinib | 4162.61 |
| lenvatinib | 2126.51 |
| lenvatinib | 6162.60 |
| lenvatinib | 4144.57 |
| lorlatinib | 6765.19 |
| midostaurin | 9211.37 |
| midostaurin | 18422.74 |
| midostaurin | 9260.04 |
| midostaurin | 18471.41 |
| nilotinib | 2893.12 |
| nilotinib | 3780.40 |
| nintedanib | 1680.06 |
| nintedanib | 3238.32 |
| osimertinib | 7582.10 |
| palbociclib | 3841.67 |
| pazopanib | 3227.01 |
| pazopanib | 1137.85 |
| pazopanib | 4248.48 |
| pazopanib | 2205.54 |
| ponatinib | 5490.78 |
| ponatinib | 6174.25 |
| ribociclib | 1847.51 |
| ribociclib | 3557.28 |
| ribociclib | 5254.62 |
| ripretinib | 16304.54 |
| ruxolitinib | 4912.60 |
| ruxolitinib | 4750.00 |
| ruxolitinib | 4798.67 |
| ruxolitinib | 4912.61 |
| ruxolitinib | 2510.53 |
| ruxolitinib | 2375.00 |
| ruxolitinib | 2423.67 |
| selpercatinib | 2137.60 |
| selpercatinib | 8062.61 |
| selumetinib | 7112.60 |
| selumetinib | 17537.60 |
| sorafenib | 4150.01 |
| sunitinib | 665.21 |
| sunitinib | 1294.65 |
| sunitinib | 1907.75 |
| sunitinib | 2533.10 |
| tepotinib | 9104.80 |
| trametinib | 7526.00 |
| trametinib | 5685.15 |
| vemurafenib | 6691.51 |
| zanubrutinib | 7932.56 |

Note: Repeats of the same drug are listed where the drug has multiple listings on the PBS, which can vary according to drug indication or whether the dispensation occurs in a public or private hospital.
